# Insight into unique somitogenesis of yak (*Bos grunniens*) with one additional thoracic vertebra

**DOI:** 10.1186/s12864-020-6598-9

**Published:** 2020-03-04

**Authors:** Yu Wang, Haoyang Cai, Xiaolin Luo, Yi Ai, Mingfeng Jiang, Yongli Wen

**Affiliations:** 1 College of Life Science and Technology, Southwest Minzu University, Chengdu, 610041 Sichuan China; 2 Center of Growth, Metabolism, and Aging, Key Laboratory of Bio-Resources and Eco-Environment, College of Life Sciences, Sichuan University, Chengdu, 610064 Sichuan China; 3 Sichuan Academy of Grassland Sciences, Chengdu, Sichuan China; 4 Key Laboratory of Sichuan Province for Qinghai-Tibetan Plateau Animal Genetic Resource Reservation and Exploitation, Chengdu, 610041 China

**Keywords:** The Jinchuan yak, Somitogenesis, Population genetics, Plateau adaptation, Molecular breeding, Marker-assisted selection

## Abstract

**Background:**

The yak is a species of livestock which is crucial for local communities of the Qinghai-Tibet Plateau and adjacent regions and naturally owns one more thoracic vertebra than cattle. Recently, a sub-population of yak termed as the Jinchuan yak has been identified with over half its members own a thoracolumbar vertebral formula of T15L5 instead of the natural T14L5 arrangement. The novel T15L5 positioning is a preferred genetic trait leading to enhanced meat and milk production. Selective breeding of this trait would have great agricultural value and exploration of the molecular mechanisms underlying this trait would both accelerate this process and provide us insight into the development and regulation of somitogenesis.

**Results:**

Here we investigated the genetic background of the Jinchuan yak through resequencing fifteen individuals, comprising five T15L5 individuals and ten T14L5 individuals with an average sequencing depth of > 10X, whose thoracolumbar vertebral formulae were confirmed by anatomical observation. Principal component analysis, linkage disequilibrium analysis, phylogenetic analysis, and selective sweep analysis were carried out to explore Jinchuan yak’s genetic background. Three hundred and thirty candidate markers were identified as associated with the additional thoracic vertebrae and target sequencing was used to validate seven carefully selected markers in an additional 51 Jinchuan yaks. The accuracies of predicting 15 thoracic vertebrae and 20 thoracolumbar vertebrae with these 7 markers were 100.00 and 33.33% despite they both could only represent 20% of all possible genetic diversity. Two genes, PPP2R2B and TBLR1, were found to harbour the most candidate markers associated with the trait and likely contribute to the unique somitic number and identity according to their reported roles in the mechanism of somitogenesis.

**Conclusions:**

Our findings provide a clear depiction of the Jinchuan yak’s genetic background and a solid foundation for marker-assistant selection. Further exploitation of this unique population and trait could be promoted with the aid of our genomic resource.

## Background

The yak is a species of livestock that is well-adapted to the extreme environment of plateaus and plays an important role in local residents’ lives. Recently, a unique sub-population was identified and referred to as the Jinchuan yak due to its higher meat yield owing to an additional thoracic vertebra [1]. For example, the average net weight of male Jinchuan yaks with 15 pairs of ribs could be over 12 kg heavier than that of male Jinchuan yaks with 14 pairs of ribs [2], demonstrating the great economic potential of the T15L5 Jinchuan yak. The normal thoracolumbar vertebral formula of yak is T14L5 (14 thoracic vertebrae and 5 lumbar vertebrae). However, several alternative formulae exist for the Jinchuan yak. It was reported that around 52% of Jinchuan yaks are T15L5, 37% are T14L5, 8% are T14L6, and 3% are T15L4 which were determined by anatomical observation [3]. The same number of thoracic vertebrae but different number of lumbar vertebrae increases the genetic heterogeneity among individuals with 15 ribs. Unfortunately, the thoracolumbar vertebral formula is often determined post-slaughter as it is hard to judge whether a live yak expresses an extra vertebra. Therefore, there is an urgent need to find a method to select individuals with more vertebrae.

A mature somite, which consists of two compartments: the sclerotome and the dermomyotome, is derived from the presomitic mesoderm (PSM) while vertebrae are derived from the sclerotome compartment [4]. The number of somites is controlled by a segmentation clock [5] and the identity of somites (vertebrae) is specified by Hox genes in the PSM [6]. A faster segmentation clock could produce embryos with increased vertebral number [7]. Substantial advances have been made in the molecular mechanisms of embryonic somitogenesis, which strongly support the investigation of a wide variety of phenotypic traits relevant to vertebrae [8].

The increased vertebral number has not only been observed in yaks, but also in cattle [9], sheep [10] and certain breeds of pigs [11–13]. Studies on multi-rib pigs date back to the early twentieth century [14]. In recent years, the methods researchers commonly take to locate regions and loci that impact the number of vertebrae include quantitative trait loci (QTL) analysis [15, 16] and genome-wide association study (GWAS) [11, 12], which require large numbers of (around one thousand) offspring derived from a few (around four) parents. One gene with considerable influence on thoracic vertebral number (TVN) in pigs was found to be Vertnin (VRTN) [11]. The biological mechanism of VRTN affecting somitogenesis has been reported to be the acceleration of the segmentation clock through the Notch signaling pathway [17].

Besides increased vertebral number, the Jinchuan yak also possesses other advantageous characteristics including high milk production [2] and an earlier age at first calving (85.4% of the Jinchuan yak calved first at three years old, which is one year earlier than average) [18], so one of the aims of this study is to dissect the population genetic characteristics of the Jinchuan yak via comparing with the Qinghai-plateau yak. Another important aim of our study is to seek markers which can be used to selectively breed Jinchuan yaks with a thoracolumbar vertebral formula T15L5. We also seek a greater understanding of the mechanisms relevant to the aberrant somitogenesis. To this end, we performed whole genome resequencing of 15 yaks whose thoracolumbar vertebral formulae were confirmed by an anatomical method to exclude the genetic heterogeneity introduced by the T15L4 and T14L6 individuals.

## Results

### Reads statistics and variants annotation

#### Sequencing and variants calling

A total of 463.64 G bases composed of 1545 million pairs of raw reads were generated on an Illumina HiSeq X Ten sequencing platform. 1522 million pairs of clean reads containing 456.61 G bases remained after filtering (Table 1). The average sequencing depths of samples reached above 10X, which ensured the accuracy of the genotypes calling. Around 17 million SNPs and 2 million InDels were called with GATK. The average variants calling rate for all individuals was 93.4% (94.3% for SNPs and 86.4% for InDels).

**Table 1:** Summary of each individuals’ sequencing information

Samples	# Raw reads	# Clean reads	Clean Base (Gb)	Average Depth
YC1	106,652,787	105,113,768	31,534,130,400	11.94
YC2	127,528,345	126,155,996	37,846,798,800	14.34
YC3	110,054,170	109,046,964	32,714,089,200	12.39
YC4	98,439,661	97,186,917	29,156,075,100	11.04
YC5	120,316,613	118,876,788	35,663,036,400	13.51
ZC1	93,795,354	92,871,350	27,861,405,000	10.55
ZC2	89,386,096	81,346,718	24,404,015,400	9.24
ZC3	98,237,486	97,483,485	29,245,045,500	11.08
ZC4	94,029,951	93,143,964	27,943,189,200	10.58
ZC5	94,768,929	93,561,321	28,068,396,300	10.63
GY1	120,612,083	118,972,444	35,691,733,200	13.52
GY2	106,477,289	105,676,535	31,702,960,500	12.01
GY3	93,962,434	93,240,641	27,972,192,300	10.60
GY4	93,469,905	92,595,105	27,778,531,500	10.52
GY5	97,742,066	96,765,431	29,029,629,300	11.00

#### Population-level genotypes and variants

**Fig. 1:**
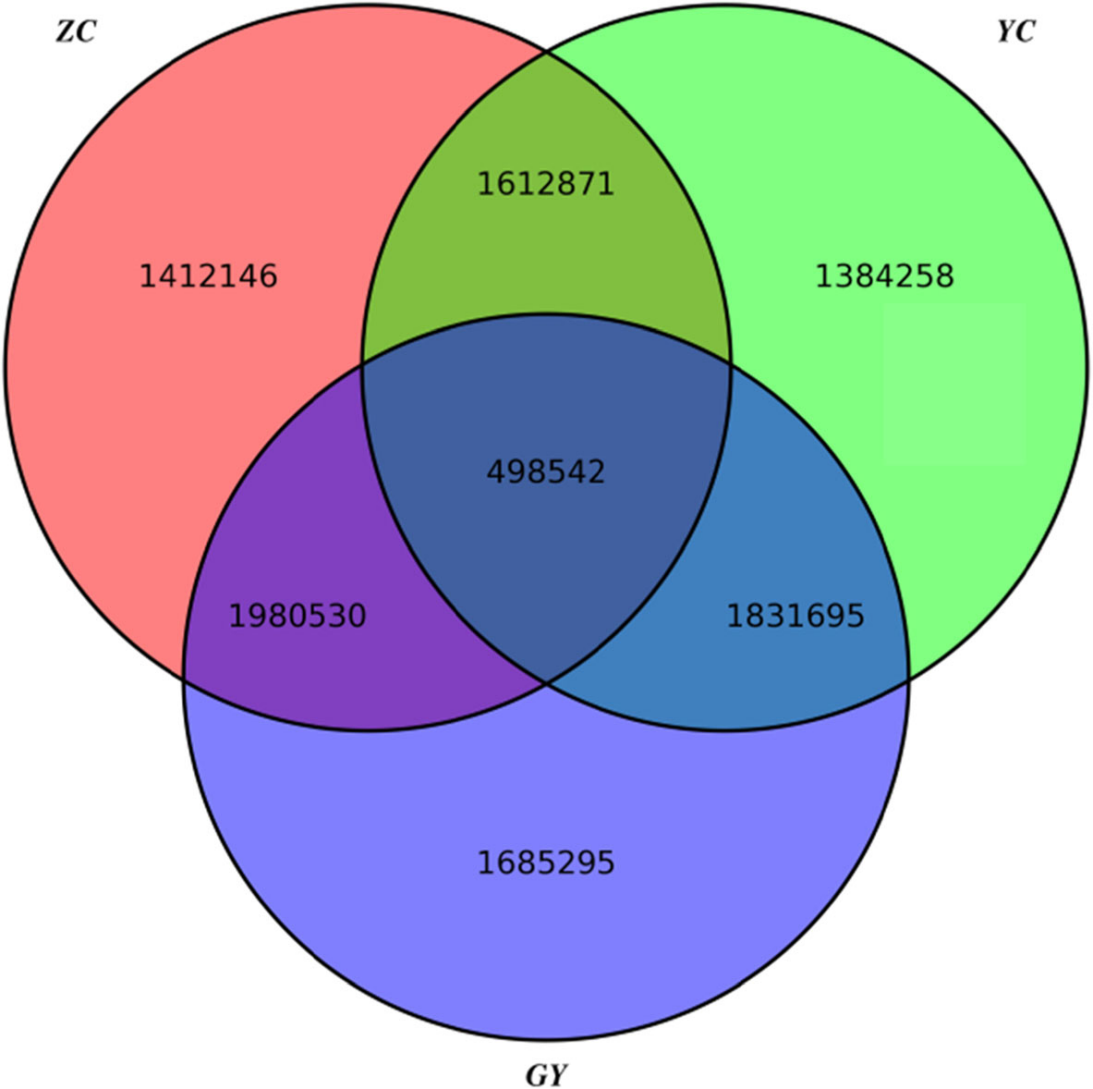
Shared loci harboring population-level genotypes among populations

As the aim of this study was to discover population-level features rather than the characteristics of any one individual, population-level genotypes were identified first. Population-level genotypes were defined as the genotypes those were the same in all five individuals in one population. Ten million out of 17 million SNPs were identified to be population-level variants in at least one population and the relationship between loci harboring these variants is shown in Fig. 1. We observed the number of loci shared between the ZC population (five Jinchuan yaks whose thoracolumbar vertebral formula was T14L5) and YC population (five Jinchuan yaks whose thoracolumbar vertebral formula was T15L5) (1,612,871) was less than the number of loci they shared with the GY population (five Qinghai-plateau yaks whose thoracolumbar vertebral formula was T14L5) (ZC-GY: 1,980,530 and YC-GY: 1,831,695). The variants of each population that resided in population-level loci were further functionally annotated (Table 2). The number of variants found in each type was similar in the three populations. The variants in exonic regions of genes hit around 5 thousand genes per population (ZC: 4670; YC: 4943; GY: 4780) and the nonsynonymous SNVs hit around 3 thousand genes per population (ZC: 3040; YC: 3257; GY: 3100). A stopgain SNV (gene14456:rna17636:exon2:c.G167A:p.W56X) and a nonsynonymous SNV (gene14456:rna17636:exon3:c.G1661C:p.R554P) were observed in the gene VRTN, which was reported to affect the somite number in pigs [11, 17]. These two SNVs were present in all individuals with an average 8X sequencing depth. A stoploss SNV (gene20267:rna24673:exon6:c.A365G:p.X122W) and a nonsynonymous SNV (gene20267:rna24673:exon6:c.C295A:p.R99S) were observed in the gene TMEM200 in all individuals with an average > 12X sequencing depth. Finally, another transmembrane protein TMEM192 (discussed in the section Association analysis) was also identified because nine population-level variants associated with the trait resided within 5 kb of the gene.

**Table 2:** Annotation of population-level SNV of each population

Values	ZC	YC	GY
splicing	93	105	96
intronic	228,602	239,609	228,036
intergenic	668,240	694,364	655,789
UTR3	3488	3662	3411
UTR5	1050	1131	1071
ncRNA_exonic	1230	1253	1168
ncRNA_splicing	1	2	1
ncRNA_intronic	2662	2628	2636
ncRNA_UTR5	2	3	3
upstream	8342	9110	8458
downstream	8360	8948	7798
upstream;downstream	221	270	245
exonic	13,084	14,769	13,097
exonic;splicing	18	18	19
nonsynonymous SNV	6451	7307	6526
synonymous SNV	4723	5266	4712
stopgain SNV	61	76	62
stoploss SNV	14	12	13
unknown	1853	2126	1803

### Population genetics analysis

#### Principal component analysis

**Fig. 2:**
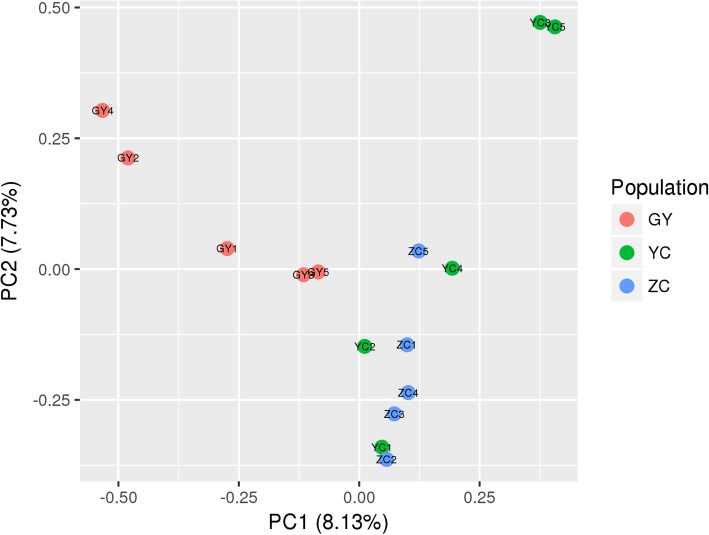
Principal component analysis

Fifteen individuals were divided into two groups (5 Qinghai-plateau yaks in one group and 10 Jinchuan yaks in another group) by the principle component 1 (PC1) (Fig. 2). The PC1 is the principal component with the greatest variance; hence this indicated that the genetic difference between the Jinchuan yak and the Qinghai-plateau yak was the greatest and the result was in line with a general expectation. However, we should also note that: (1) the proportion of the information contained in the PC1 (8.13%) and the PC2 (7.73%) was very close; (2) the division of individuals according to the PC2 was different from that according to the PC1. It reflected the genetic variance that the PC2 represented accounted for a large proportion and was different from what the PC1 represented. Interestingly, the manner that PC2 separated individuals was nearly the same as our phenotypic grouping manner, which suggested the genetic variance represented by the PC2 might overlap with the genetic variance impacting the thoracolumbar vertebral formula. As no principle component separated individuals into two groups corresponding to the YC population and the ZC population well, the region regulating the trait was putatively a small proportion of genomic regions.

#### Linkage disequilibrium

**Fig. 3:**
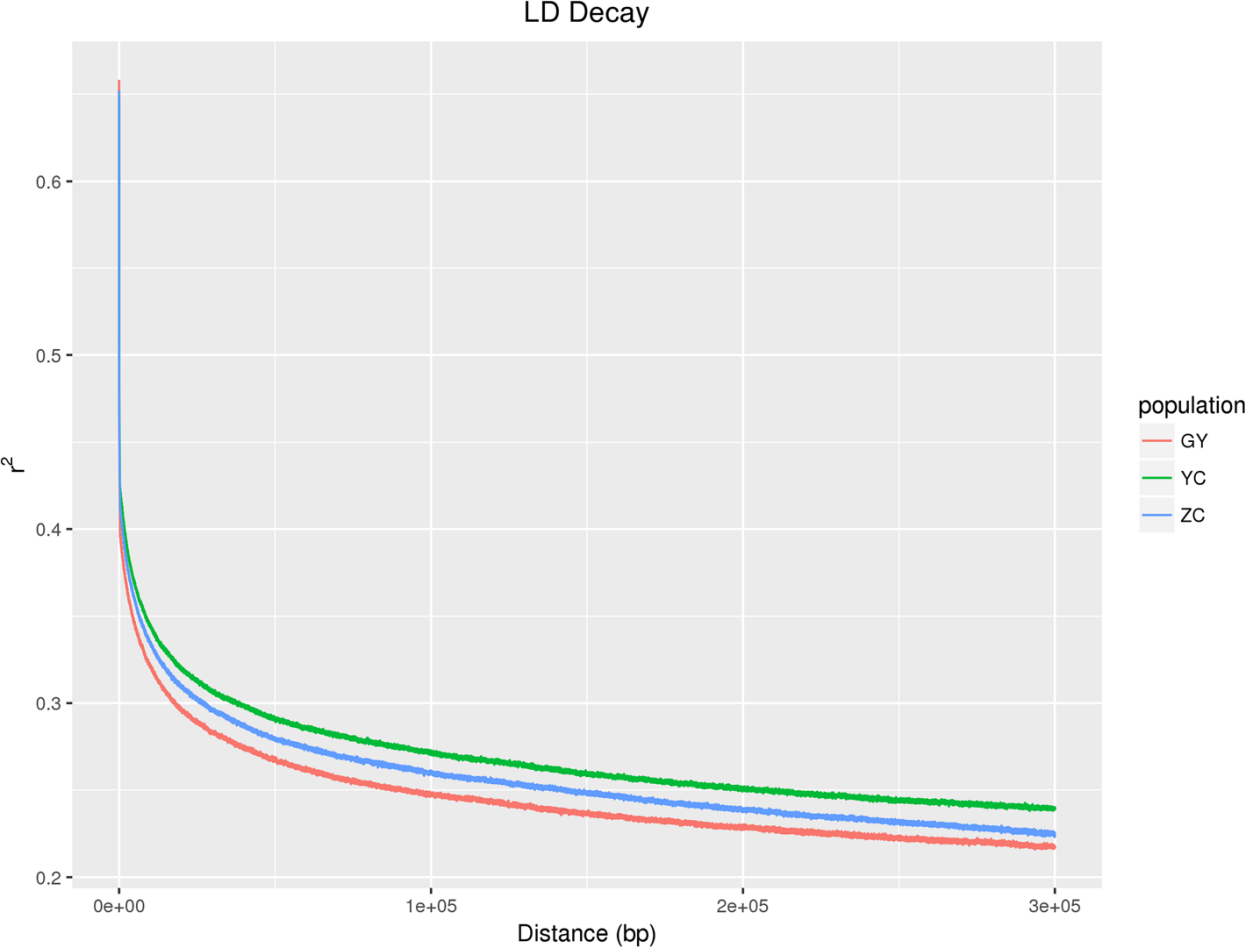
Decay of linkage disequilibrium

The slowest decay rate and the highest level of linkage disequilibrium (LD) were observed in the YC population, whereas the rapidest decay rate and the lowest level of LD were observed in the GY population (Fig. 3).

This phenomenon can be attributed to the intense artificial selection for the extra thoracic vertebra trait in heavier yaks which may lead to the decline in genetic diversity and increased linkage among loci. This was also likely reflected in the effective population size (*N*_*e*_) for the YC population which was the smallest compared with the ZC and GY populations. Due to the slow LD decay rate of the YC population, it became less difficult to select loci linked to the causal variants determining the trait of increased vertebral number for selective breeding.

#### Phylogenetic tree

**Fig. 4:**
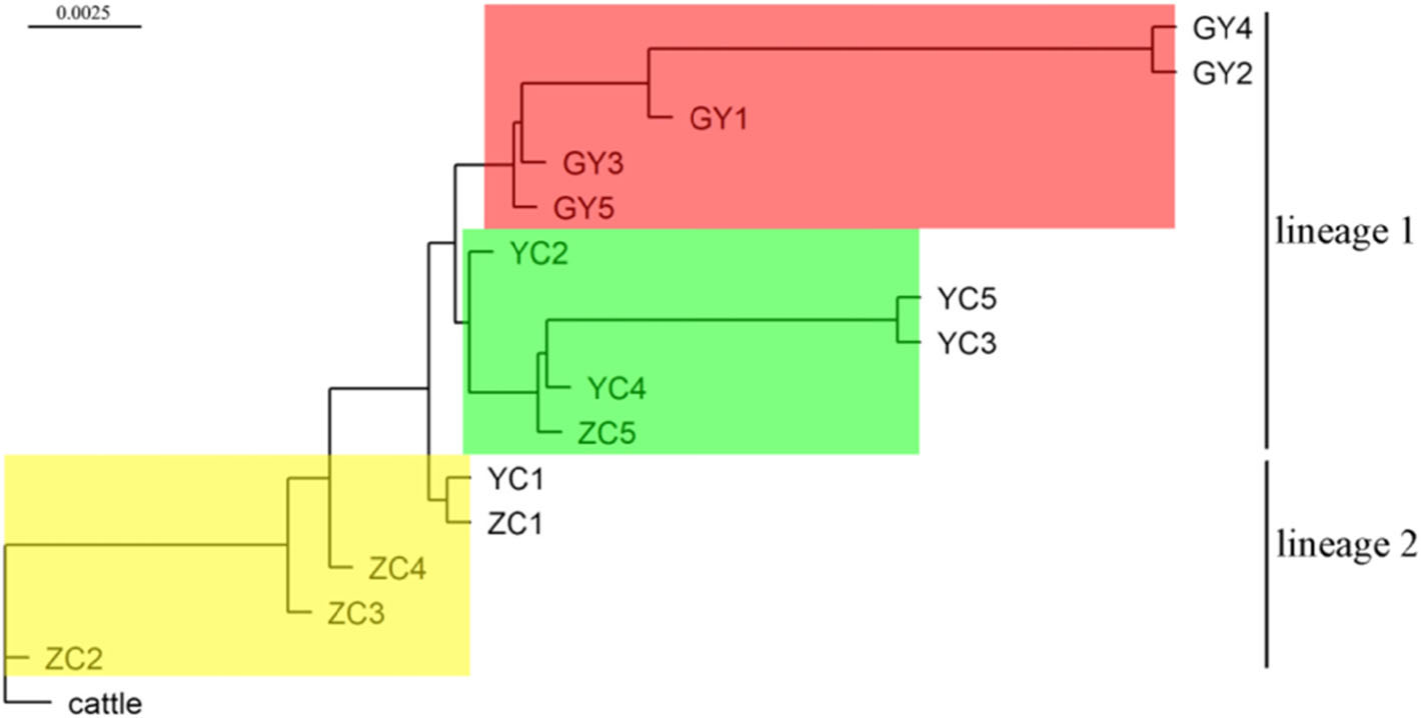
Neighbor-join tree constructed using (1-IBS) distance between animals. Cattle was used as an outgroup. Those with close (1-IBS) distances were highlighted with a block with the same color. Of note, the bar of the plot only represented distances between the nodes except tips. The unit distance from a tip to its corresponding closest node was different from the bar. The distance of the cattle’s tip to its corresponding closest node was 0.33 and the average distance of all other yaks’ tips to their corresponding closest nodes was 0.11. The purpose of adjusting the unit distance was to better display the distance between individuals and blocks. The lineage to which each animal belongs was labeled on the right of the tree

The NJ (neighbor-joining) tree split individuals into three groups, which are highlighted with blocks of different colors (Fig. 4). It was reasonable to sort five Qinghai-plateau yaks into one group (highlighted with a red block) because these individuals have lived in an environment that is different from Jinchuan yak’s for a long time.

However, the large genetic distance within Jinchuan yak suggested a significant genetic diversity exists in the Jinchuan yaks. Considering the genetic distance between Jinchuan yaks, Qinghai-plateau yaks and the outgroup (cattle), it suggested that Jinchuan yaks could be further divided into two subpopulations, which were highlighted with a green block and a yellow block. The genetic distance from the green block to the red block was closer than to the yellow block. And the yellow block was closer to the outgroup cattle than any other blocks were.

In light of the previously reported phylogeographical study of domestic and wild yaks based on complete mitochondrial sequences [19], it was suggested that the animals highlighted in the yellow block were from one lineage (referred to as lineage 2 to make the nomenclature to be consistent with the previous research [19], the same below) because of its closer proximity to the outgroup cattle, and those highlighted in the green block were from another lineage (referred to as lineage 1, the individuals constitute this lineage are widely distributed around Qinghai-Tibetan Plateau) due to its closer proximity to the red block than the yellow block. The numbering of the lineages here was consistent with the nomenclature used in the phylogeographical study [19], which concluded that three different lineages evolved allopatrically and then reunited into one gene pool before the start of domestication. Four individuals from ZC population were from lineage 2. And four individuals from YC population and all five individuals from GY population were from lineage 1. This suggests that variants determining the trait were inherited from a common ancestor of lineage 1 and were further fixed in YC population, which resulted in 52% of Jinchuan yak with one additional thoracic vertebra [3]. Therefore, the markers we identified here may still be useful to select those originating from common ancestors in other places.

Another interesting observation was that the genetic distances between individuals shown in the NJ tree were similar to the distances between individuals reflected by PCA (Fig. 2), such as the close distances between GY2 and GY4 and between YC3 and YC5. However, the relationship between ZC1 and YC1 presented in the PCA was not as close as it was in the NJ tree. These differences are reasonable to observe because the (1-IBS) distance matrix was calculated using 14 million whole genome SNVs, but the variances of PC1 and PC2 only contributed 15.86% to the total variance. We also observed that the relationships presented in the NJ tree were closer to what PC2 reflected, where 5 individuals from the GY population and 3 individuals from YC populations were located above the y = 0 line and 4 individuals from the ZC population located below the y = 0 line. In other words, PC2 grouped individuals from the GY and YC populations into a cluster and individuals from the ZC population into another cluster which was consistent with the result of the NJ tree. This suggests that the similarity inherited from the same ancestors was second only to the similarity that gradually accumulated through living in the same environment. In summary, the results of PCA and phylogenetic analysis indicated that two lineages existed in our samples, which contrasted with their classification based on the differences in morphologies and geographical distribution.

#### Regions under selective pressure

The nucleotide diversity (pi) and population-differentiation statistic (Fst) were calculated using whole genome SNVs. The average pi of each population (YC: 0.00134, ZC: 0.00130, GY: 0.00123) was similar to other domestic yaks’ pi that was also calculated from whole genome sequencing data (unselected landraces (D2): 0.00138, Tianzhu white yaks (D1): 0.00137, 36]. The average Fst between Jinchuan yaks and Qinghai-plateau yaks was 0.0415; this was larger than the Fst (0.0213) between D1 and D2 but smaller than that (0.0582) between D1 + D2 and wild yaks [20], which indicated that a relatively high population differentiation exists between the Plateau yak (represented by Qinghai-plateau yaks) and the Valley yak (represented by Jinchuan yaks). The pattern identified in our study was similar to that found by others using the same type of data which indicated that our data truly reflected the genetic characteristics of the objects we selected.

**Fig. 5:**
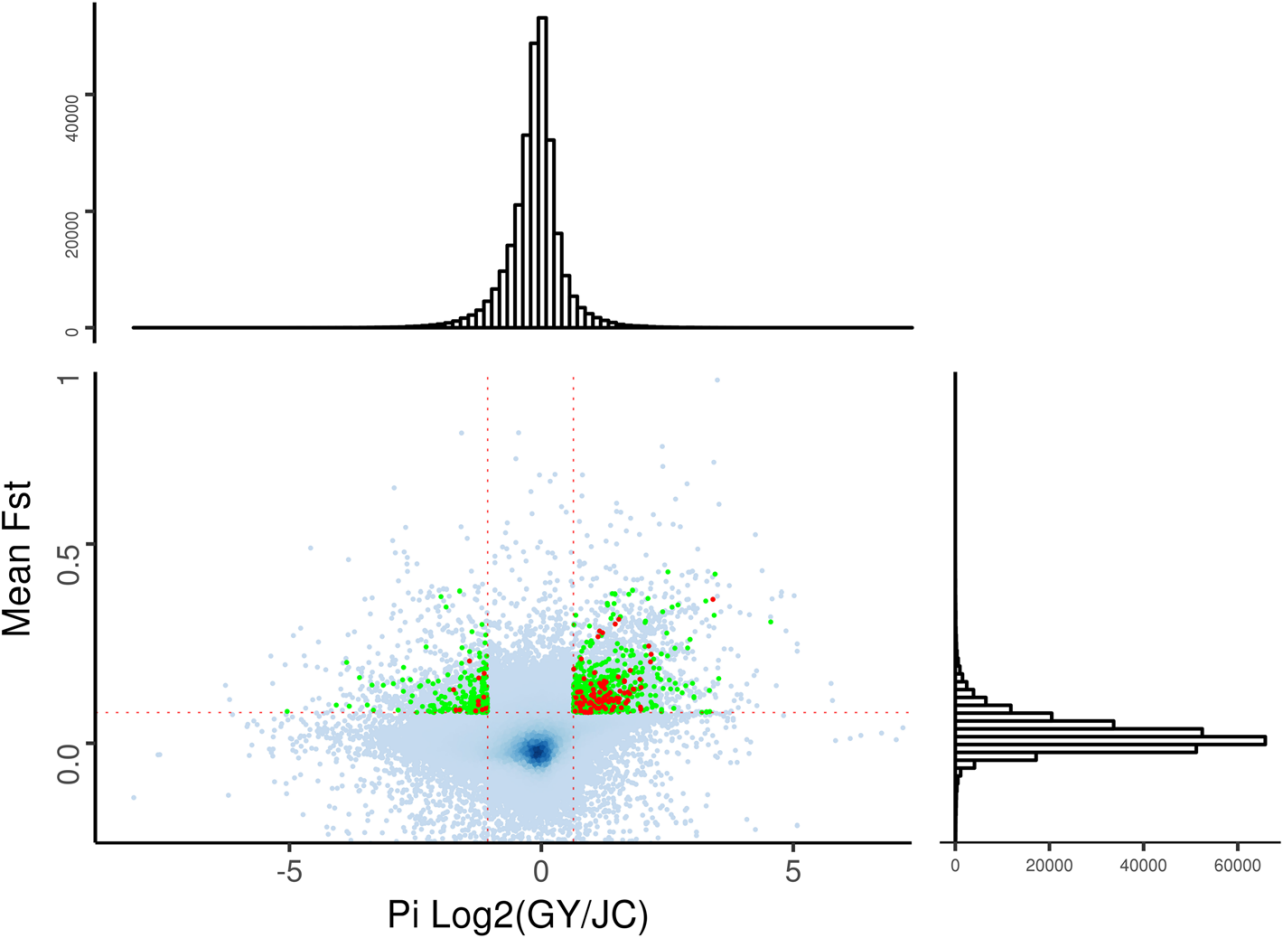
Selective sweep analysis. In this plot, the darker the shade of the blue points, the greater the number of points present at that location. Horizontal and vertical red dotted lines are used to split the top 5% of points from all other points. The points (windows) with the top 5% Fst and log2 (pi ratio) simultaneously were colored green if they reside in a gene. Furthermore, the points were colored red if the genes they reside in were enriched in a biological terms. The upper and right histograms indicate the number of points in the corresponding intervals

To explore the source of the generation of an extra vertebra and advantageous productive traits of Jinchuan yaks from an evolutionary perspective, selective sweep analysis was conducted between Jinchuan yaks and Qinghai-plateau yaks. Qinghai-plateau yaks were chosen as the control group because they belong to Plateau yak and Jinchuan yaks belong to Valley yak. The genetic distance between them should be relatively far away. The 5 kb regions whose Fst and pi log-ratio were both in the top 5% were regarded as regions under strong selective sweeps. 1393 windows covering 30.8 Mb genomic regions and 4154 windows covering 62.5 Mb genomic regions were chosen for the GY population (which refers to 5 Qinghai-plateau yaks) and the JC population (which refers to 10 Jinchuan yaks), respectively. The size of the regions under selective sweep in the Qinghai-plateau yak was less than half that of the Jinchuan yak. A possible reason for this phenomenon was that the habitat of the Qinghai-plateau yak seldom changed but the habitat of the Jinchuan yak might have undergone a major change. The genes that overlapped with these regions (S1 Table) were identified as the candidate genes under strong selective sweep. Furthermore, six genes (DMD, GPC6, KLF12, MAGI2, NXPH1, and TTC13) were identified in both populations because they overlapped with different windows having different pi log-ratio. The regions overlapping with genes are represented as green points in Fig. 5. A notable phenomenon revealed by our data was that the green points were generally located more internally relative to the blue points, which didn’t overlap with genes but were under stronger selective sweep than the green points were. It suggested that the regions overlapping with genes were more stable than the regions that were likely to be functionless.

The genes under strong selective sweep were used to perform enrichment analysis in DAVID for the JC and GY populations. The enriched terms are listed in Table 3. Among the genes under selective sweep for the JC population, 27 genes (*COL4A6, COL2A1, COL12A1, MEP1A besides genes whose name beginning with ‘LOC’*) were involved in the ‘protein digestion and absorption pathway’, 14 genes (*ERBB4, ADCY3, ADCY1, P2RX1, PDE1C, CCKBR, HRH1, PLCB1, ADRA1A, RYR2, ATP2A3, GRM1, LOC102275229, LOC102287375*) were located in the ‘calcium signaling pathway’, 10 genes (SLC1A7, SLC38A1, PLCB1, ADCY3, ADCY1, GRM1, GRM8, GRIK3, GRIA3, GRIA4) resided in the ‘glutamatergic synapse pathway’. In short, pathways related to two types of functions were significantly enriched: one was related to energy (‘protein digestion and absorption’, ‘alpha-linolenic acid metabolism’ and ‘pancreatic secretion’) and the other was related to domestication (‘calcium signaling pathway’, ‘p53 signaling pathway’, ‘glutamatergic synapse’ and ‘retrograde endocannabinoid signaling’). For the GY population, only two pathways related to the cell junction were found to be enriched, which were adherens junctions and tight junctions. The regions under strong selective sweep were related to totally different functions in the two populations, which suggested these two populations underwent different adaptive evolution and it was consistent with their different geographical distribution.

**Table 3:** Enrichment analysis of the genes hit by the top 5% of points

Population	Category	Enriched Term
JC (Valley yak)	KEGG	Protein digestion and absorption
	KEGG	Ether lipid metabolism
	KEGG	alpha-Linolenic acid metabolism
	KEGG	Glutamatergic synapse
	KEGG	p53 signaling pathway
	KEGG	Calcium signaling pathway
	KEGG	Glycosaminoglycan biosynthesis - chondroitin sulfate / dermatan sulfate
	KEGG	Retrograde endocannabinoid signaling
	KEGG	Melanogenesis
	KEGG	Pancreatic secretion
	KEGG	Prolactin signaling pathway
	KEGG	Circadian entrainment
	GO_MF	calcium ion binding
	GO_MF	NN-dimethylaniline monooxygenase activity
	GO_CC	organelle membrane
	GO_CC	extracellular region
	GO_BP	lipid catabolic process
GY (Plateau yak)	GO_MF	ligase activity
	GO_MF	isomerase activity
	KEGG	Adherens junction
	KEGG	Tight junction

### Marker-assisted selection

#### Identify 330 candidate markers associated with the T15L5 trait

The screening of loci associated with the T15L5 trait started from 498,542 loci harboring population-level genotypes (Fig. 1). Among them, 330 loci were selected as candidate markers for selective breeding because the population-level genotypes of these loci of the YC population were different from those of the ZC-GY population. A total of 61 genes harboring these loci or being within 5 kb of these loci were identified as possible causal genes of the T15L5 trait. The top 5 genes carrying the most loci were PPP2R2B (25 [harboring these loci] + 0 [within 5 kb of these loci]), TBL1XR1 (23 + 1), SHB (10 + 2), TMEM192 (0 + 9) and two genes (BPIFB3, 5 + 1 and PCP4L1, 0 + 6). According to the reports, PPP2R2B [21] and TBL1XR1 [22, 23] were most likely to be related to the T15L5 trait (the relationship between these two genes and the development of somites are depicted in the discussion section).

The selection of a specific trait is equivalent to the selection of the causal alleles/genotypes which manifest this trait. The alleles/genotypes that neighbor causal ones would also be selected together due to their associated linkage. Therefore, the number of candidate markers on each chromosome was determined and, as a result, the top 3 chromosomes carrying the most markers were chromosome 1 (39 loci, harboring TBL1XR1), chromosome 17 (35 loci, harboring TMEM192) and chromosome 7 (31 loci, harboring PPP2R2B) (Fig. 7). The genes carrying the most loci were also located on these three chromosomes, which indicated that QTLs determining the T15L5 trait of the Jinchuan yak were likely linked with or located within these genes.

A comparison of the sequences of the PPP2R2B gene from yak, cattle (*Bos taurus*) and goat (*Capra hircus*) uncovered a fact that the PPP2R2B gene sequence of yak was assembled into two contigs separately. The PPP2R2B genes from cattle and goat are comprised of eleven and ten exons respectively, and the total lengths are 509,525 bp and 478,425 bp. But the length of yak’s PPP2R2B gene is 129,107 bp which is made up of the last eight exons (including introns between these eight exons). The distance between cattle’s first three exons (the head part, denoted by H) and the last eight exons (the tail part, denoted by T) was 184,756 bp and the total length of the last eight exons (including introns between these eight exons) was 128,197 bp. Moreover, the corresponding distances in goat were 188,301 bp (between first two exons (H) and last eight exons (T)) and 130,901 bp (total length of the last eight exons including introns). The distant distance between H and T might be the reason for the loss of H in the yak, which may result from artificial annotation and not the intrinsic nature of this gene in yak. The similar lengths and sequences of the last eight exons between yak, cattle, and goat indicated the correctness and reliability of the annotated part of yak. No population-level variants were observed in the head part of yak’s PPP2R2B gene. The reference genome of the latter two species has already reached the chromosome level but the reference genome of yak was still fragmented, which reminded us of the necessity of improving the yak’s reference genome for further understanding the molecular mechanism underlying the yak’s good characteristics.

#### Definition and selection of best markers

**Fig. 6:**
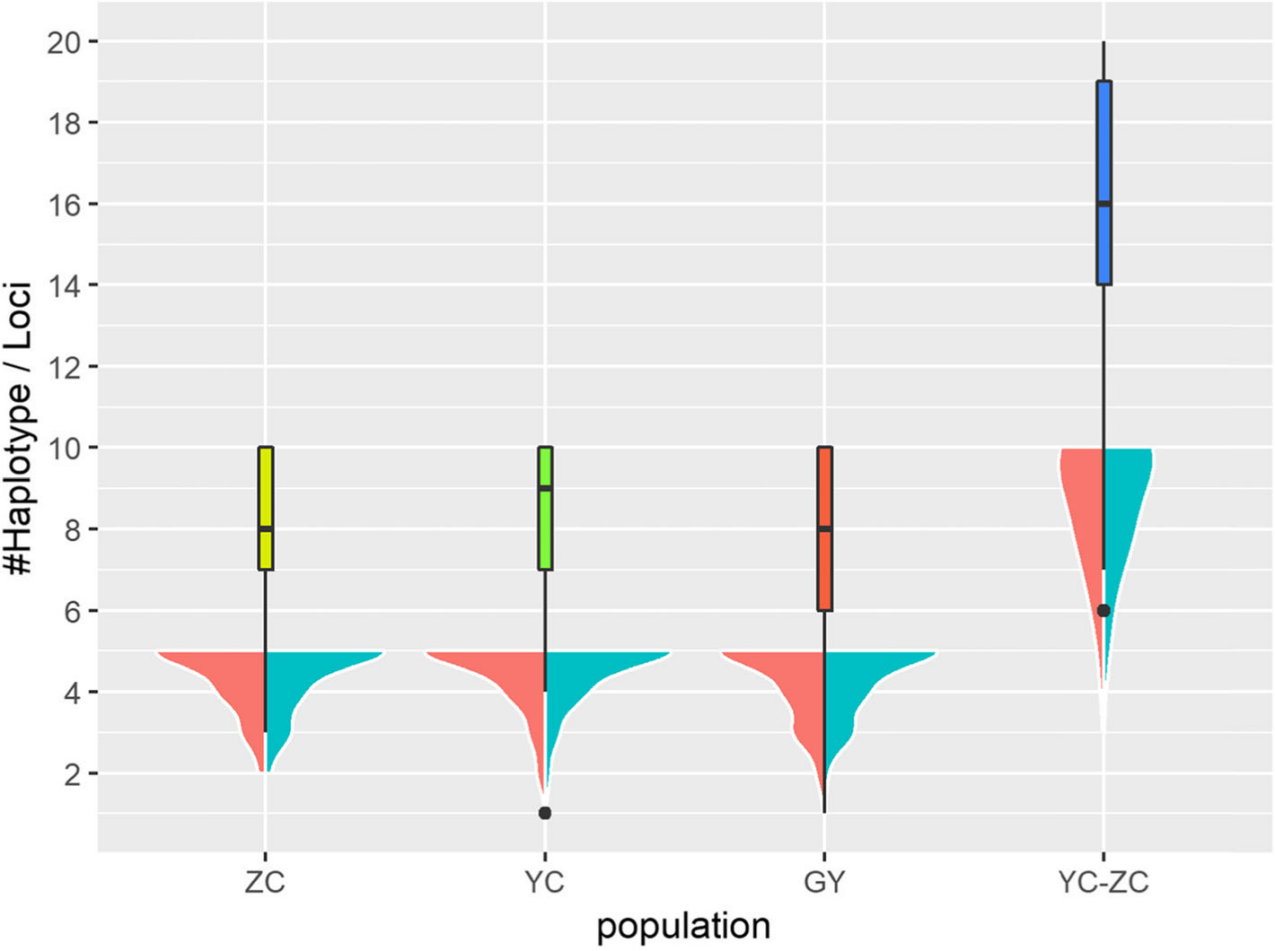
The violin plot of the haplotype diversity. The boxplot of each population is shown according to the total number of haplotypes for each locus. The corresponding density plot of each population is composed of two parts. The two parts were asymmetry and separately represented the distribution of the number of the haplotypes of loci on one side

**Fig. 7:**
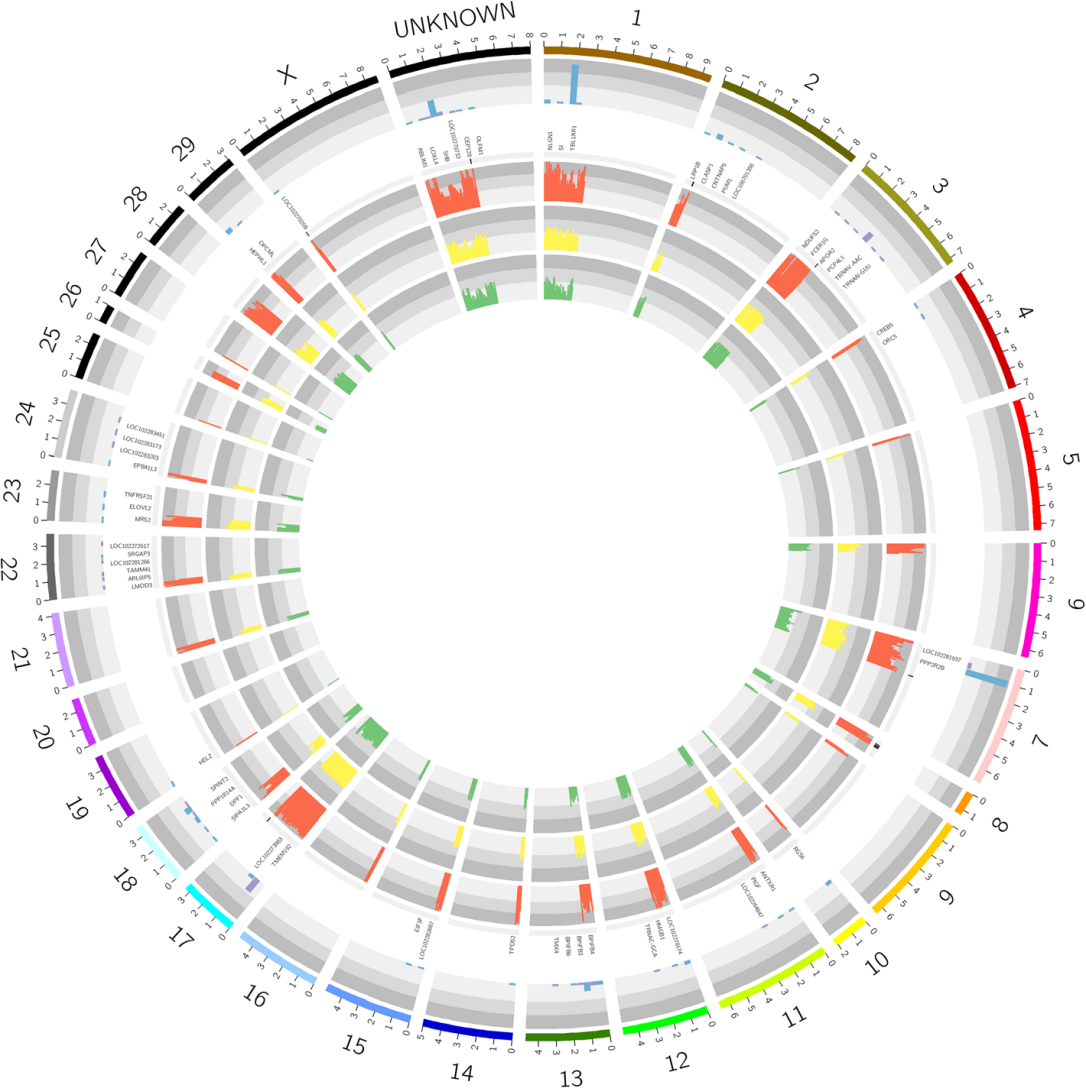
Seven factors were considered in the selection of the 10 most reliable markers. Each factor corresponds to one of seven circles. A number from 1 to 7 was assigned to each circle from the outside to the inside. The first circle represents 29 autosomes, 1 X chromosome and 1 extra chromosome used to carry the contigs that were not assigned to chromosomes. The third circle is representative of the gene names; while the histogram shown in the second circle aligned with the third circle which represented the number of loci in this gene. Each gene corresponded to two bars: the left blue bar indicated the number of loci resided in this gene and the right purple bar indicated the loci located at the outside of the gene but within 5 kb of the gene. The 4th, 5th, 6th and 7th circles were aligned with each other and represented the conditions of each locus. The locus whose genotype was homozygous is shown as a black line in the fourth circle. The number of haplotypes for a locus was represented by the length of this locus’ bar at the 5th, 6th, and 7th circle corresponding to the YC-ZC population, the YC population and the ZC population, respectively

For developing markers used to select individuals with the T15L5 trait, a small portion of loci were further isolated from the 330 loci and validated in 51 extra samples. The three primary factors considered during selection were the haplotype diversity of a locus, the genes and the chromosomes where loci resided (Fig. 7) and the combination of alleles (genotype) of loci (heterozygous or homozygous). For the haplotype diversity, the genotypes of loci residing within 5 kb of 330 loci were extracted into 330 groups and each group of genotypes was phased with Beagle separately. For each locus, two haplotypes would be constructed. The haplotype diversity of a locus for a population was defined as the count of distinct haplotypes of this locus in this population. The haplotype diversity of each population is presented in Fig. 6. 25% of loci represented 20 different haplotypes in the YC-ZC population and should be avoided because of their high haplotype diversity. Another 25% of loci representing no more than 14 different haplotypes were preferred due to their low haplotype diversity. The YC population was observed to have the highest haplotype diversity derived from these 330 loci because it had the largest median and the least loci with low haplotype diversity. It likely indicated that Jinchuan yaks with the T15L5 trait had relatively high genetic diversity in these loci.

For the genes and chromosomes (Fig. 7), those harboring the most loci were preferred so the loci that resided in the genes (PPP2R2B, TBL1XR1, and TMEM192) and the chromosomes (1, 7 and 17) had a high priority. For the combination of alleles of loci, the loci with homozygous genotypes for the YC population were preferred because the genotypes of progeny whose two parents had the same homozygous genotypes would be the same with their parents. The ratio of homozygous loci to heterozygous loci was 17:313. According to the criteria mentioned above, 13 loci were selected to be genotyped in 51 extra Jinchuan yaks with known thoracolumbar vertebral formula.

#### 7 best markers validated by target sequencing

Considering the cost of further validation, a stepwise strategy was adopted to alleviate the bias introduced by population stratification. Ten loci were selected to be genotyped in 26 Jinchuan yaks (including the 15 resequenced samples used as the standard). According to the concordance between the genotypes of 15 resequenced samples obtained from the whole genome resequencing and the target sequencing, 3 loci were excluded. Therefore, 3 extra loci from the 330 candidate loci were chosen. The new ten loci were genotyped in 50 Jinchuan yaks (including the 10 resequencing samples). In summary, 51 extra Jinchuan yaks were genotyped with target sequencing and the evaluation of these 7 loci which appeared in two rounds of target sequencing is presented in Tables 4 and 5.

**Table 4:** The evaluation of the ability of 7 loci predicting 20 vertebrae

diff > 1	observed 19	observed 20	total	accuracy of prediction
expected 19	24	4	28	85.71%
expected 20	4	2	6	33.33%
unknown	13	4	17	
total	41	10	51	
representative capacity	58.54%	20.00%

**Table 5:** The evaluation of the ability of 7 loci predicting 15 thoracic vertebrae

diff > 4	observed 14	observed 15	total	accuracy of prediction
expected 14	18	4	22	81.82%
expected 15	0	2	2	100.00%
unknown	23	4	27	
total	41	10	51	
representative capacity	43.90%	20.00%		

The evaluation consisted of two aspects: one was the ability to successfully predict 20 thoracolumbar vertebrae and the other was to predict 15 thoracic vertebrae. First, the loci whose major genotypes differed in two phenotype groups (e.g. the ‘observed 19’ group and the ‘observed 20’ group) were selected. The major genotypes denoted the genotype whose count was the most in one locus. Second, each individual was scored for two phenotypes according to all its selected genotypes and the rule of scoring was: + 3 if the genotype probability > = 0.9; + 2 if the genotype probability > = 0.8 and + 1 for the rest. Third, if the difference of scores between two phenotypes (diff) was greater than a specific value, this individual was considered to be predictable using our loci and its phenotype was specified as the phenotype corresponding to the higher score. Others are specified as unknown. Finally, the efficiency of properly predicting a phenotype was assessed with ‘accuracy of prediction’ and ‘representative capacity’. The accuracy of prediction was defined as the percentage of individuals with a correctly determined phenotype relative to the total individuals who were predicted to be this phenotype. The definition of representative capacity was the percentage of individuals with a correctly determined phenotype relative to the total individuals who were observed to be this phenotype.

In the evaluation of the ability of these 7 loci to predict 20 thoracolumbar vertebrae, 6 out of 51 individuals were expected to be 20 thoracolumbar vertebrae and 2 out of 6 were observed to be 20 thoracolumbar vertebrae. Therefore, it indicated the accuracy of predicting 20 thoracolumbar vertebrae was 33.33%. And 2 out of 10 individuals with 20 thoracolumbar vertebrae were identified, which indicated the representative capacity was 20%. When it came to predicting 15 thoracic vertebrae, the accuracy increased to 100.00% but the representative capacity was still 20.00%. One reason that could explain the difference between the accuracies of two conditions was the difference between 19 and 20 vertebrae was smaller than the difference between 14 and 15 thoracic vertebrae. The representative capacity told us the 10 Jinchuan yaks resequenced before only represented around 20.00% of the total genetic diversity of individuals with T15L4/T15L5 traits.

## Discussion

The objective of this study was to explore the genetic background of the Jinchuan yak and the possible mechanisms of aberrant somitogenesis according to the whole genome resequencing data in order to find markers which could be used to select individuals with the specific thoracolumbar vertebral formula (T15L5) trait. The representative capacity (20.00%) was satisfying in our view considering our relatively small initial sample size and the use of only 7 markers to predict the trait. In the future, more loci could be included to increase the representative capacity of the genetic diversity at least in theory. After identifying the causal gene(s) or QTLs dominate for this trait, more precise selection could be carried out using the corresponding markers.

**Fig. 8:**
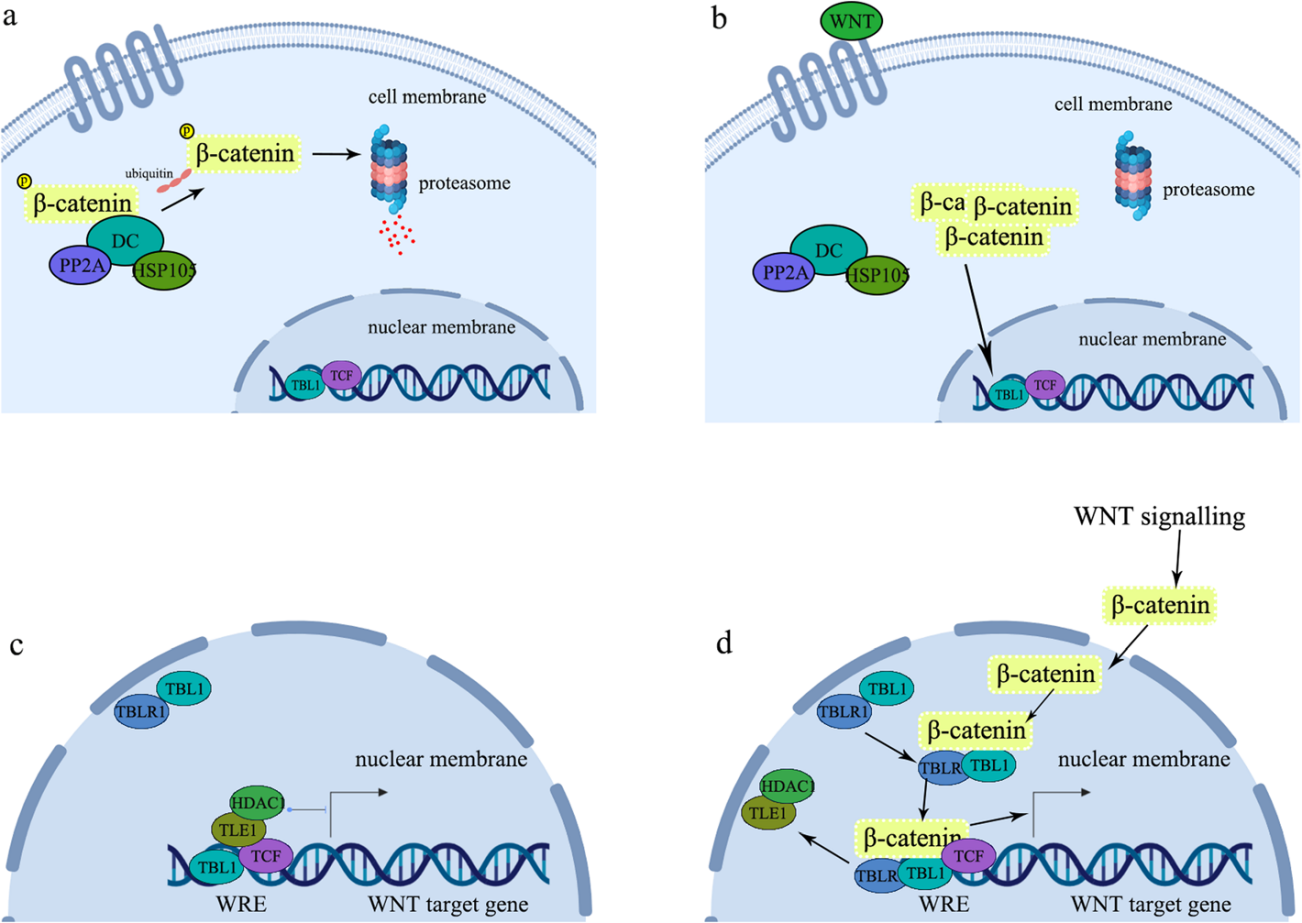
Role of PP2A and TBLR1 in WNT/beta-catenin signaling pathway. **a**. beta-catenin is phosphorylated by the degradation complex (DC) with PP2A and HSP105 as its components, and it undergoes phosphorylation-dependent ubiquitination without the stimulus wnt and degraded by proteasome. **b**. The accumulation of beta-catenin induced by the stimulus wnt. **c**. The co-repressors TLE1 and HDAC1 repress the wnt target genes. **d**. TBL1-TBLR1 and beta-catenin recruit each to remove co-repressors TLE1 and HDAC1 and activate the transcription of wnt target genes. This figure is our own

The predominant type of PP2A is a heterotrimer phosphatase, which consists of a scaffolding subunit A, a regulatory subunit B and a catalytic subunit C [24], and one of the substrates it could bind is APC [25], which is also the component of the beta-catenin degradation complex. Four gene families exist for the B subunit and the PPP2R2B gene identified in our study is in the PPP2R2 gene family [24]. PP2A complexes contain different regulatory B subunits that exist in disparate tissues at varying times [24]. PP2A is able to be recruited by the beta-catenin degradation complex carrying the component HSP105 to antagonize the phosphorylation of beta-catenin and prevent beta-catenin from phosphorylation-dependent ubiquitination, which leads to wnt-induced beta-catenin accumulation in the cytoplasm [26] (Fig. 8 a & b). Msgn1 is reported to function as a direct target gene of Wnt3a performing the duty of transferring the spatial signal from the Wnt3a/beta-catenin gradient to the Notch signaling program to control the segmentation clock during somitogenesis [21]. The period of the segmentation clock is regulated by intercellular coupling through the Notch signaling program and disruption of this coupling has been reported to increase the period of zebrafish somitogenesis and its embryonic somite length [27]. A faster segmentation clock and embryos with longer and shorter body segments were observed in transgenic zebrafish lines with elevated Notch signaling caused by a high copy number of DeltaD [7]. Altogether, the mutation of PPP2R2B gene was inferred to be the cause of the increased vertebral number of the Jinchuan yak. The variants of the PPP2R2B gene were primarily located in introns, similar to another study that also identified one genetic variant in the intron of the PPP2R2B gene and found it to be associated with altered breast cancer risk and recurrence [28].

Another important gene identified in this study that was found to carry numerous mutations was TBL1XR1 (also known as TBLR1), which was both involved in the signaling pathways regulating somite identity and somite number. The relationship between TBLR1 and somite identity originated from the corepressor SMRT [22]. TBLR1 is reported to be a core component of SMRT (silencing mediator of retinoic acid and thyroid hormone receptors) [23]. The establishment and maintainence of the somitic Hox code and segmental identity during embryonic development requires the SMRT-dependent repression of RAR (retinoic acid receptors) [22]. The ratio of thoracic to lumbar vertebrae changed from T13:L6 to T14:L5 in mice whose SMRT function in NR signaling was specifically disabled [22]. Therefore, the mutation of the TBLR1 gene might influence the normal function of SMRT and hence the segmental identity. As for the relationship between TBLR1 and the somite number, the necessity of mutual recruitment between TBLR1 and β-catenin for the activation of the transcription of the wnt3a target genes [29, 30] (Fig. 8 c and d) indicated that the mutations in TBLR1 we observed here might influence the original function of β-catenin and hence the somite number as with the PPP2R2B gene.

When a species evolves under a certain pressure, the areas harboring alleles that help the species better adapt to this pressure would be under strong selective sweep. For example, the windows in which the SOCS2 and GPX3 genes reside were screened out with selective sweep analysis of *Ovis aries* based on sequencing data similar to ours. The functions of these genes did help the species to better adapt to their corresponding environments [31]. In our study, no genes were screened out under very strong selective sweep between Plateau yaks and Valley yaks. It might be explained by the fact that no extreme difference exists in the ecological environments between the Tibetan Plateau and the surrounding Hengduan mountains because they are both at an altitude of at least 2000 m [1].

## Conclusions

The increased vertebral number of yak in a natural condition is believed to be relevant to its adaption to plateau. Investigating the mechanism determining this trait and developing methods selecting such individuals would provide us better understanding of the mechanism regulating this trait and much more production yields. Our work provides a good practice of implementing MAS with the aid of modern NGS-based molecular methods to select yaks with one more thoracic vertebra. The report of the unique somitogenesis of yak and the discussion of possible mechanisms underlying this trait will inspire further exploration concerning the somitogenesis and plateau hypoxia. The interpretation of population genetics characteristics of this unique sub-population of the yak builds a foundation for both selective breeding and evolutionary development. Also, the genomic resources of individuals whose thoracolumbar vertebral formula was confirmed by anatomical observation in our research provide researchers with a reliable foundation to carry out further comparative analysis.

## Methods

### Grouping of samples and sequencing

In order to explore the genetic characteristics of Jinchuan yaks with one additional thoracic vertebra, five Jinchuan yaks whose thoracolumbar vertebral formula was T15L5 (YC population) were taken as the case group and ten yaks with a normal vertebral number (five Jinchuan yaks (ZC population) and five Qinghai-plateau yaks (GY population) whose thoracolumbar vertebral formula was T14L5) were taken as the control group. Our samples could also be divided into another two groups according to a classical classification, which categorizes them as Plateau yaks (represented by the Qinghai-plateau yak) and Valley yaks (represented by the Jinchuan yak here; also named JC population) based on their geographic distribution and morphological features. Therefore the genetic characteristics at the population level of the Valley yak relative to the Plateau yak were also explored in this study. All study animals were slaughtered on breeding farms and the number of thoracic and lumbar vertebrae of each yak was determined to confirm its thoracolumbar vertebral formula. Ten Jinchuan yaks and five Qinghai-plateau yaks were from Jinchuan Yak Breeding Cooperative in Reta Village, Maori Township, Jinchuan county, Sichuan province and Qinghai Yushu Plateau Yak Farm in Yushu county, Qinghai province respectively. Whole blood was collected from each animal and used to extract genomic DNA. Pair-end libraries were constructed according to the protocol of the manufacturer (Illumina, San Diego, CA, USA) using the extracted genomic DNA. All samples were sequenced on an Illumina HiSeq X Ten sequencing platform.

### Reads alignment and variant calling

The raw pair-end reads generated after sequencing were filtered out under the following conditions to generate clean reads: (a) It contained a sequencing adapter; (b) The length of ‘N’ bases in either of a pair-end reads exceeded 10% of the total bases of the corresponding read; or (c) Low quality bases (Q-score < = 5) in either of a pair-end reads that accounted for over 50% of total bases of the corresponding read. The clean reads were then mapped to the yak reference genome (BosGru_v2.0 [32]) with the BWA-MEM (v0.7.15) [33] using the parameter ‘-a’. The alignment results were further filtered using Picard (v2.5, http://broadinstitute.github.io/picard/) to remove duplicates with ‘MarkDuplicates REMOVE_DUPLICATES = true’ and samtools [34] to remove records with ‘view -F 3584 -q 30’. Variant calling was performed with the tools ‘HaplotypeCaller’ and ‘GenotypeGVCF’ in GATK (v3.6) [35]. ANNOVAR was used to annotate variants [36].

### Population genetic analysis

The principle component analysis (PCA) was performed based on the whole genome SNVs of each individual using the program smartpca provided in EIGENSOFT (version 7.2.1) [37]. The squared correlated coefficient (r^2^) of paired SNVs at certain distances was calculated using PopLDdecay [38] for each population. LD decay was visualized using ggplot2 according to r^2^ and its corresponding distance.

The pairwise (1-IBS) distance (IBS: identity-by-state) matrix between individuals was calculated with whole genome SNVs using PLINK [39] and used to construct a neighbor-joining (NJ) tree using PHYLIP [40]. The visualization of the tree was implemented by ggtree [41]. Cattle (*Bos taurus*) (NGS data accession ID: ERR035712 - ERR035769), whose whole genome SNVs were obtained according to the same method we used for our samples here, was selected as an outgroup to root the NJ tree.

Both the nucleotide diversity (pi) of the Jinchuan yak (composed of the ZC and the YC population) and Qinghai-plateau yak (the GY population) and the fixation index (following Weir and Cockerham’s paper [42]) were calculated using vcftools [43] with a sliding-window method (50 kb windows and a 10 kb step length). The pi ratio (pi of GY/pi of JC) was log2-transformed to generate a “log2 (pi ratio)”. The enrichment analysis of the candidate genes that were covered by the windows with the top 5% Fst and log2 (pi ratio) simultaneously were implemented with DAVID [44]. All yak annotated genes (BosGru_v2.0 [32]) were used as the background to displace the default background provided by DAVID.

### Marker-assisted selection

The yak reference genome (BosGru_v2.0 [32]) we used in this study was aligned to a chromosomal level assembly of the yak reference genome (Accession ID: PRJNA435474. This chromosomal level assembly was obtained by scaffolding the reference genome we used here in virtue of the reference genome of closely related species cattle.) with LASTZ [45] to anchor scaffolds to their possible corresponding chromosomes. The genotypes of loci residing within 5 kb of the 330 loci were extracted into 330 groups and phased with Beagle [46] to help us evaluate the haplotype diversity of the region where each locus resided. Seven factors that we considered when we selected the most reliable candidate markers were illustrated with CIRCOS [47]. Thirteen loci out of 330 loci were picked out stepwise and target sequencing of these loci was carried out with Illumina NextSeq500. Primers used to amplify the sequences harboring the final seven loci are presented in S2 Table. All 51 extra Jinchuan yaks were from the same place as the ten Jinchuan yak. The reads generated here were mapped to the reference genome to call variants and generate the genotypes of these loci.

## Supplementary information

**Additional file 1.** The genes that overlapped with regions under strong selective sweeps.

**Additional file 2.** Primers used to amplify the sequences harboring the final seven loci.

## Data Availability

The datasets generated and/or analysed during the current study are available in the NCBI repository (https://www.ncbi.nlm.nih.gov/) and are available under the accession number PRJNA516576.

## Abbreviations

Fst: Population-differentiation statistic
GWAS: Genome-wide association study
IBS: Identity by state
kb: Kilo-base pair
LD: Linkage disequilibrium
m: Meter
MAS: Marker-assisted selection
NGS: Next-generation sequencing
NJ: Neighbor-joining
PC: Principal component
PCA: Principal component analysis
pi: Nucleotide diversity
PSM: Presomitic mesoderm
QTL: Quantitative trait loci
RAR: Retinoic acid receptors
SMRT: Silencing mediator of retinoic acid and thyroid hormone receptors
SNP: Single nucleotide polymorphism
SNV: Single nucleotide variant
Tn1Ln2: The number of thoracic vertebrae is n1 and the number of lumbar vertebrae is n2
TVN: Thoracic vertebral number
